# Evaluation of production of xanthan gum utilizing the corn cob liquor as a carbon source in different strains of *Xanthomonas campestres*

**DOI:** 10.1186/1753-6561-8-S4-P211

**Published:** 2014-10-01

**Authors:** Meirielly Jesus, Petherson Araújo, Tamara Silva, Elisiane Reis, Denise Ruzene, Daniel Silva, Francine Padilha

**Affiliations:** 1 Universidade Tiradentes, Aracaju, SE, Brasil; 2 Universidade Federal de Sergipe, SE, Brasil

## Background

The xanthan gum production has been growing significantly. It is estimated an annual increase by about 5 to 10%. The production needs a carbon source in fermentation media and are commonly employed as glucose or sucrose, which accounts for about 50% of the cost of production. Therefore the use of agro-industrial residues, such as corn cob for the production of xanthan gum becomes suggestive, as such residues are abundant. Waste when processed becomes a rich source of carbon, nutrients and salts. Thus this study aims to use hemicellulose fractions, derived from agro-industrial residues.

## Methods

The cobs were selected, dried at room temperature and submitted to grinding in knives mill to 16 mesh and then alkaline extraction was performed utilizing 0.75 mol NaOH for 120 minutes, and then filtered and stored on cooling (-20°C) until used [1]. The strain was acquired bank Xanthomonas Institute of Technology and Research, where four strains of Xanthomonas campestris (629, S6, 254 and 1078) are stored in YM medium consisting of (gL-1): extract yeast 3.0, malt extract 3.0, peptone 5.0, sucrose 10.0, and agar 20.0, the microorganism being incubated at 28 º C for 24 hours. The fermentation process was performed in two steps, the inoculum was prepared by adding 1 ml of bacterial suspension 108 in 14 ml of YM medium incubated on an orbital shaker at 150 rpm, 28 ° C for 24 hours. During the second stage, the inoculum was added to 86mL of fermentation media. The media were evaluated for carbon sources, and the presence of salts. The nutritive media were: M1 (liquor corn cob), M2 (liquor corn cob + salts), M3 (sucrose), M4 (sucrose + salts). After inoculation, was incubated at 180 rpm for 96 hours at 28 ° C [2]. For each experimental condition was performed four replicates, which was analyzed productivity and apparent viscosity. Subsequent to the fermentation broth was centrifuged at 4700 xg for 40 minutes to separate cells. The biopolymer is precipitated with alcohol at 92.6% (1:4 v / v) and followed by drying in an oven at 50 ° C for 24 hours. The samples were dialyzed against water for 24 hours, changed every 6 hours, and subsequently lyophilized and evaluated the apparent viscosity. The viscosity was measured from a 3% aqueous solution at shear rate of 0-300 s^-1^.

## Results

The results show that the strains analyzed showed similar behavior as the production of the polymer in the four conditions of fermentation, which emphasized the S6 strain, which exhibited the highest performance in its production in all conditions including in the absence of salts, which the sample M1 obtained 11,7038g.L^-1 ^.h^-1 ^and M2 obtained 6,486475 g.L^-1^.h^-1 ^biopolymer. It can be concluded that the results achieved demonstrated the feasibility of obtaining gum in media with the hemicellulose fractions, enabling greater feasibility of process within the context of a biorefinery. The apparent viscosity of aqueous solutions has shown that the use of the corn cob liquor has improved the viscosity of the polymer obtained.
